# The Effect of Temperature on the Distribution of Zoonotic Pathogens in Livestock and Wildlife Populations: A Systematic Review

**DOI:** 10.1155/2023/2714539

**Published:** 2023-08-18

**Authors:** Zoe A. Becvarik, Kayla S. Smurthwaite, Aparna Lal

**Affiliations:** National Centre for Epidemiology and Population Health, Research School of Population Health, Australian National University, Canberra, ACT, Australia

## Abstract

**Background:**

Evidence for the impact of climate change on the distribution of zoonoses has largely focussed on the burden in humans and is lacking information on the effect of temperature on nonvectorborne zoonoses that are transmitted indirectly through contaminated environments. We present a systematic literature review on the impact of temperature on the distribution of zoonotic pathogens in mammalian livestock and wildlife populations, with a focus on nonvectorborne zoonoses that can be spread through air, water, food, and soil.

**Methods:**

We systematically searched PubMed, Scopus, and Web of Science, as well as grey literature, and screened titles, abstracts, and full text. English, peer-reviewed, and full text studies were included if they: focused on temperature; considered incursion, distributional burden or risk; and focused on a zoonotic pathogen in livestock and/or wildlife populations of mammalian vertebrates that can be transmitted through indirect pathways without a nonmammalian and nonvertebrate intermediate host.

**Results:**

Temperature was an important determinant of zoonoses distribution across all 17 studies included in the final review, with 11 studies finding a positive association. The majority of studies focused on parasites (7) and bacteria (9) and were conducted in the northern hemisphere. Two studies provided future climate projections that identified areas of increasing prevalence and expanded risk for pathogens that were already established. However, no studies specifically investigated the risk of zoonotic incursion with increasing temperature. Few studies explored how local variations in temperature and urbanisation interact with distal changes like Arctic warming to affect the distribution and spread of nonvectorborne pathogens through food, water, and soil.

**Conclusions:**

The review's findings point to the value of a One Health approach to biosecurity that builds on the interconnected relationship between human, animal, plant, and environmental health. Such research is urgently needed to inform the prioritisation and risk assessment of zoonoses more comprehensively in a rapidly changing climate.

## 1. Introduction

**Figure 1:**
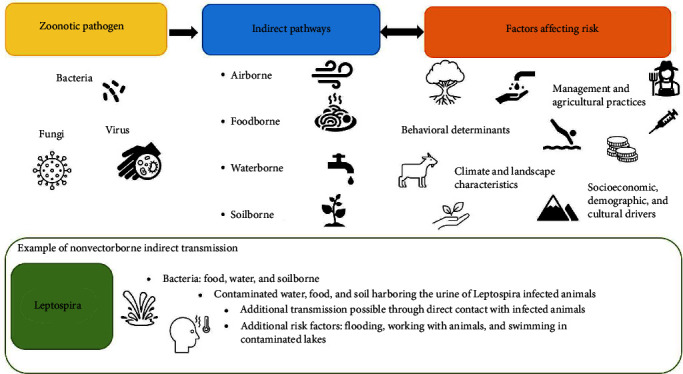
Conceptual diagram of alternative pathways of indirect transmission that are not vectorborne. The diagram depicts indirect transmission pathways of different zoonotic pathogens, such as bacteria and parasites, and the more complex relationship with additional factors that affect risk.

Zoonotic pathogens comprise ∼60% of all known human infections and 75% of emerging infectious diseases, with many being sensitive to the changing climate [1–6]. Climate change poses a significant threat to air, food, water, and soil security [7], leading to new opportunities for the spread of zoonotic pathogens through these pathways [8, 9] and resulting in a substantial threat to the economy and public health [10, 11]. The incursion (i.e., the introduction or new establishment) of pathogens that cause disease in animals can compound population vulnerabilities and negatively impact community wellbeing [11]. For example, leptospirosis has a high burden in human populations in Southeast Asia and also has significant implications for animals, and therefore, livestock productivity and food security [12, 13]. Thus, zoonotic incursions pose a significant biosecurity and global health risk with climate change [3, 11, 14]. To date, there is a little understanding of how climate change will impact the spread and incursion of pathogens that are not transmitted through vectors (e.g., mosquitoes), but through contaminated air, food, water, and soil [15, 16] (Figure 1).

The impact of temperature changes on the spread of pathogens in livestock and wildlife that have the potential to cause serious illness in humans is understudied compared to the impact on diseases that already have a substantial burden in humans. A recent review showed that rising temperatures may cause poleward expansion and seasonal changes in zoonotic diseases [17], a finding echoed by a second review of neglected tropical diseases (many of which are zoonotic) which found that temperature contributed to a shift in geographic distribution [18]. For example, a shift in the distribution of African trypanosomiasis toward the East African highlands was predicted, where an incursion would have a substantial impact on economic activity and food security, as well as exacerbate spill-over into humans [18]. Such reviews, however, have focused primarily on diseases that already have a substantial burden in humans, such as vectorborne zoonoses.

Recent incursions of zoonotic pathogens that have received significant public attention have involved mammalian vertebrate animals as reservoir hosts, such as Monkeypox and Japanese Encephalitis Virus (JEV) [2, 19, 20, 21]. Mammalian vertebrates have also been implicated as important intermediate hosts for global zoonotic transmission [22]. Understanding how temperature, as a key component of climate change, can influence the interaction between pathogens, the environment and mammalian vertebrate hosts at different points in the transmission cycle is critical to predicting future biosecurity and health threats. Furthermore, although there is an increasing recognition of the complex and interconnected role of various climatic and nonclimatic factors, the Intergovernmental Panel on Climate Change Sixth Assessment Report [23] provides greater impetus for this systematic review as it highlights the increasing risk of climate-related environmental and health impacts as temperatures continue to rise. The aim of this review was to systematically identify and assess literature that examines the impact of temperature on the incursion and distribution of zoonotic pathogens that are transmitted through indirect pathways and exclude nonmammalian and nonvertebrate intermediate hosts.

## 2. Methods

This review follows the Preferred Reporting Items for Systematic Review and Meta-Analyses (PRISMA) Guidelines [24]. A protocol for this study is currently being assessed for publication in Biomed Central Systematic Reviews and is published online using Open-Science Framework.

### 2.1. Eligibility Criteria

Studies were included based on the criteria in Table 1. Incursion was defined as the introduction of a pathogen into a new geographic area and distribution referred to the relative burden of the pathogen on the local livestock and/or wildlife population, as defined by its temporal and/or spatial distribution. According to the WHO [25] definition, zoonotic diseases were considered to be any disease or infection that is naturally transmissible from vertebrate animals to humans through direct or indirect transmission. Diseases were considered for inclusion in this review if there was sufficient evidence to suggest that they were natural transmissible from vertebrate animals to humans, even if this did not occur with great regularly.

### 2.2. Information Sources

Our search strategy employed medical subject headings (MeSH) terms, as well as keywords as per the search strategy in Appendix S1. We searched PubMed, Scopus, and Web of Science on the 17^th^ of August 2022. A search of relevant grey literature was also conducted, as outlined in Appendix S2. A preliminary search of the formal literature was completed during March and April 2022.

### 2.3. Search Strategy

The specific search strategy employed in the systematic review included the search terms outlined in Appendix S1. The search was conducted by two reviewers (ZB and KS). The keywords used in the grey literature search (see Appendix S2) were developed in accordance with the formal search strategy. Furthermore, the expected suitability and frequent use of keywords such as “zoonosis” and “animals” were confirmed based on a recent bibliometric analysis relating to climate change [26]. Forward and reverse screening was conducted using the Connected Papers software [27].

### 2.4. Selection Process

The results of this systematic review were compiled using EndNote and then uploaded to the Covidence platform and duplicates were removed. Two reviewers (ZB and KS) independently screened titles and then abstracts based on the eligibility criteria. Studies that were relevant based on the eligibility criteria following abstract screening were then included in a full-text review. The full-text review was completed independently by two reviewers (ZB and KS). The reason for exclusion of studies was recorded in Covidence and disagreements between reviewers were resolved through discussion.

### 2.5. Data Extraction

Extraction of the data was conducted individually by ZB. Data were extracted for study characteristics, pathogen/s and population/s, climactic factors, nonclimatic factors, limitations, and confounders (see Appendix S3 for full extraction template). Data on other climatic factors and nonclimatic factors were extracted as this has been recognised as a key limitation of past reviews, wherein the authors were limited in the inferences they could make regarding seasonality and localised meteorological disease impacts [18]. The primary outcome of interest was the impact of temperature on the incursion or distribution of zoonotic pathogens in livestock and wildlife populations of mammalian vertebrate animals. Secondary outcomes included the impact of other climactic variables, such as humidity and precipitation.

### 2.6. Risk of Bias Assessment

The risk of bias of the included articles was assessed by study design using the Johanna Briggs Institute critical appraisal tool (Appendix S4) [28].

### 2.7. Data Synthesis and Management

**Figure 2:**
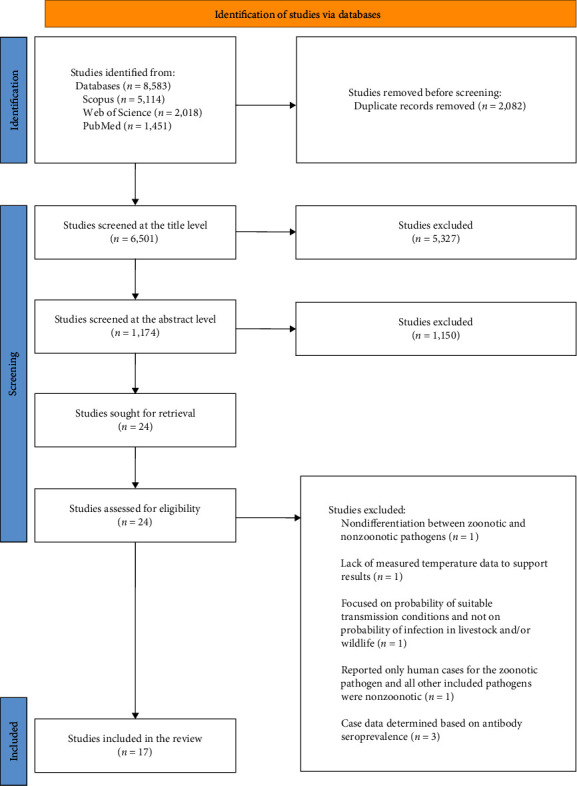
Preferred Reporting Items for Systematic Review and Meta-Analyses (PRISMA) flow diagram. PRISMA [24] flow diagram depicting the identification and selection process of included studies.

This systematic review qualitatively synthesised the included articles to communicate the key findings and summaries of the qualitative synthesis are presented in Table 2. This systematic review included a PRISMA flowchart (Figure 2), which outlines the article selection and screening process, as well as justification of exclusion. Given the heterogeneity in methodology, temperature measures, pathogens, and outcomes assessed in the included studies, we were unable to conduct a meta analysis as intended.

## 3. Results

**Figure 3:**
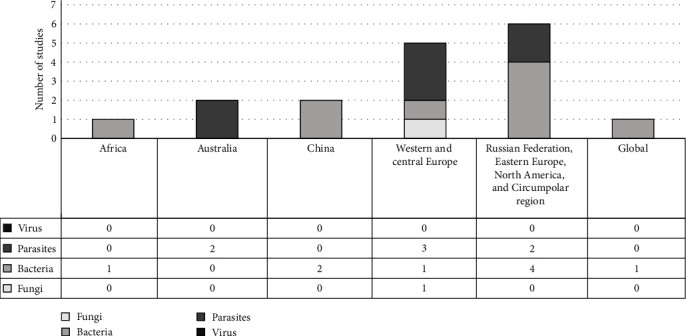
Distribution of included studies by latitude and pathogen type. Graphical representation of the number of studies reported for different geographical regions and the number of different pathogen types identified. Grouping of geographic regions was based on latitude.

The study selection process yielded 17 studies for inclusion in the final review (Figure 2). The papers included in this review were diverse in terms of methodology, pathogen and outcomes studied, as well as the region of interest. All papers were derived from the original database search with no additional literature included from the grey literature or forward and reverse screening. The studies were all cross sectional in design and employed varying modelling components, with many producing outputs through spatial analysis (see Appendix S5). Most of the studies were published in the past decade and there was a higher number of papers for the northern latitudes (Figure 3). Studies included parasites, bacteria, and one fungus (see Figure 3) and the specific pathogens within these overarching categories were also diverse (see Table 2).

Temperature was an important determinant in all included studies (Table 2). However, the association was pathogen-specific and occurred in both positive and inverse directions (see Table 3) with a range of temperature metrics being used. Positive associations with temperature were found for, *Uncinaria stenocephala*, *Ascaris suum*, *Pasteurella multocida*, *Anthracis bacillus*, *Erysipelothrix rhusiopathiae*, *Trichostrongylus columbriformis*, *Leptospira*, and *Dermatophytes*. Inverse associations with temperature were found for *Echinococcus multicocularis*, *Trichostrongylus vitrinus*, *Trichinella britovi*, *Campylobacter jejuni*, and Shigella toxin-producing *E. coli* (STEC). Some studies had less definitive results. For example, while an inverse relationship existed for *Campylobacter jejuni*, these results were not consistent across all years studied [38]. The study on *Strongyloids sp*. and *trichostronglyidae* as broad categories reported no statistically significant associations with temperature [33].

In total, 11 studies provided information on spatial variation (see Appendix S5). Overall, trends in distribution were in line with the observed effect of temperature for a given pathogen. In general, this produced spatial heterogeneity based on climate zones, as was observed in the study on *Pasteurella multocida* wherein 75% of cases originated from six ‘hot-spot' regions in Mainland China and few cases occurred in the remaining 25 provinces [37]. Some studies only analysed the effect of temperature on seasonal trends. For example, one study identified monthly trends in regional variation of prevalence of *trichostrongylosis*, *strongyloidosis*, and *nematodiris* which reflected disease seasonality, and therefore temperature more indirectly [32]. Two of the included studies also modelled future projections of the spatial distribution of zoonoses based on estimated climatic conditions and identified future areas of increased prevalence, as well as expanded risk zones [41, 42].

Three of the studies investigated the association between temperature and infection intensity [29, 31, 33]. The two studies on *Echinococcus multicocularis* found an inverse association between temperature and infection intensity and another study found a positive association for protozoan infection intensity, however, this did not differentiate zoonotic protozoans.

All studies investigated the relative contribution of other climatic, landscape, socioeconomic, or agricultural factors, in addition to temperature (see Appendix S5). Commonly reported climatic factors for which significant associations with certain pathogens were found included precipitation, rainfall, humidity, and windspeed. In the case of *Ascaris suum*, humidity and rainfall were positively associated with infection and for *Pasteurella multocida*, an inverse association was observed with wind speed [43]. Notably, humidity was seen to complicate the effect of temperature in the case of dermatophytosis in farmed rabbits [45]. Dermatophyte prevalence was significantly greater in areas with high temperature associated with humidity ranging from 62% to 65% and authors concluded that neither temperature nor humidity alone could account for the variability in prevalence observed but that both were critical to dermatophyte growth. Seasonality was also commonly assessed and observed in study analyses. For example, in the case of *Leptospira* in livestock in the Russian Arctic, there was a pronounced seasonality of disease in the spring and summer period which correlated with two significant peaks in incidence [41]. Important observations relating to socioeconomic and agricultural factors included some of the following: livestock and human density was positively associated with greater leptospirosis prevalence [41]; contact between livestock and wild areas as well as different livestock being housed together were positively associated with greater prevalence of STEC [36] and investment in agriculture was inversely associated with the prevalence of leptospirosis [41].

## 4. Discussion

In recent years, there has been an exponential growth in the number of articles studying the impact of altered climatic conditions on changing zoonotic pathogen and disease dynamics [2, 3, 15, 16]. However, there is a continued focus on vector-borne pathogens and studies of zoonoses well established in humans. To our knowledge, this is the first comprehensive review of the impact of temperature on nonvectorborne zoonotic pathogens in livestock and wildlife that are transmitted indirectly through contaminated food, water, and soil. Our review of 17 included studies highlights critical gaps in current research that include the lack of evidence on the impact of increasing temperature on the distribution of zoonoses and the risk of incursions, with only two studies considering future climate change scenarios; and how local variations in temperature and urbanisation interact with distal changes (e.g., warming in the Arctic) to affect the distribution of pathogens through air, food, water, and soil. In addition, it should be noted that the review included no studies on the impact of temperature on indirectly transmitted nonvector borne zoonotic viruses in livestock and wildlife. This may reflect the focus of existing research on zoonotic pathogens once they are established in human populations. Given the potential severity of conditions such as Lassa Fever (a haemorrhagic fever virus transmitted through aerosolised rodent excrement and contaminated food and materials), and the influence of a changing climate on habitat suitability of the rodent reservoir of Lassa Fever [46–49]; the impact of temperature on viral zoonoses in livestock and wildlife is a key gap in current literature. We identify this as a priority area for future research.

Higher temperatures are projected to increase rates of infectivity and disease burden in the immediate future for some parasites, while decreasing the potential for spread of others [50, 51]. Helminths may be particularly sensitive to climate change due to the involvement of free-living stages that are strongly influenced by temperature and moisture [50, 51]. For example, the sensitivity of Echinococcus multicocularis eggs to high temperatures and desiccation has been documented in animals and humans, with a study of human infections suggesting that this may partially explain why alveolar echinococcus occurs in the cooler parts of the northern hemisphere [29, 31, 52–54]. High temperatures also reduce Trichinella larva survival in host carrion, reducing the likelihood of consumption by scavenging hosts and further transmission [55]. It has been suggested that Ascaris lumbricoides may become the dominant helminth in Asia due to its ability to endure hotter temperatures and higher aridity [50, 56–58]. Its capacity to survive in urban environments also elicits concern regarding potential higher rates of human ascariasis that may be seen in warming Asian megacities [50, 59]. A better understanding of the role of increasing minimum temperatures and the role of urbanisation on zoonotic risk through food, water, and soil is needed.

Predominantly, prevalence and distribution of bacterial and fungal pathogens show a positive association with temperature. Gao et al. [37] suggested that the positive association between temperature and *Pasteurella multocida* may be partly explained by the relationship between heat and the immune response of affected animals because increased temperature does not immediately affect swine immunity and the mean monthly temperature and humidity of the previous month had a greater influence on infections. Heat may cause changes in adrenal hormones which inhibit humoral and cell-mediated immune responses in animals, however, evidence for this is limited [37, 60–62]. Similar to parasites, temperature affects the survival and reproduction of *Erysipelothrix rhusiopathiae* (optimum temperatures ranging from 30 to 37°C) and *Leptospira* (>7.1 or <34°C), although the increased activity of rodents during warmer periods may also drive the likelihood of exposure to L*eptospires* [63–66]. Dermatophytes are climate sensitive, preferring warm, moist body sites with their activity being greatest at 30–40°C [67–69]. Indeed, increasing ambient temperature and humidity has been suggested as a key driver of the increasing prevalence of human dermatophytosis in India over the last two decades [67, 70]. In the case of Anthracis bacillus, warming in the Arctic has resulted in thawing of permafrost and the release of anthrax spores from wild and domestic ruminant carcasses buried in the permafrost [71, 72]. Furthermore, climate induced changes in the Arctic have altered the migration route of indigenous pastoralist communities and the migratory patterns of wild ungulate species, potentially leading to new environmental pathways for spread and pathogen range expansions [6, 73, 74].

Comparison between human and animal studies is complicated because climate-driven modification of human activity and behaviour may introduce additional risks, a pattern observed for bacterial foodborne pathogens [36]. For example, the inverse association of temperature with STEC are contradicted by another animal study [75] and an Italian study in children, which found that STEC infection was positively associated with the number, duration and frequency of heat waves [76]. Similarly, the *Campylobacter jejuni* study in broiler flocks and humans found a strong positive association between temperature and *Campylobacter* infections, with the greatest effect observed at maximum weekly temperatures above 13°C [77]. The study, however, demonstrated an inconsistent relationship across locations and years studied, with a key limitation being the limited temporal scale of temperature data considered, and the exclusion of humidity, an important determinant of *Campylobacter* transmission amongst birds [38, 77, 78]. This inconsistent relationship also highlights varying relationships with different temperature metrics, the main reason that we could not conduct a meta analysis.

Although this review indicates that temperature is a key determinant of the temporal and spatial distribution of zoonotic pathogens in livestock and wildlife, evidence also highlighted the multidimensional risks to agriculture, development and livelihoods, and global health through complex and interacting pathways. Changing landscapes will have implications not just for distribution of pathogens, but also for the mode of transmission. For example, although higher temperatures may lead to increased prevalence of *Leptospira*, glacial retreat in the Himalayas is expected to contribute to increased flooding risk which could increase the likelihood of waterborne transmission [79–81]. This also highlights the need to consider the interaction between temperature, disease, and other environmental factors. In the Russian Arctic, budgetary investment in agriculture was identified as one of the most important determinants of the distribution of leptospirosis [41] and agricultural practices, such as housing multiple livestock species together and allowing livestock contact with wild areas, were associated with higher odds of STEC infection [36]. Understanding how upstream drivers such as warming in the Arctic interact with local farm management practices to drive patterns of risk for zoonotic pathogens remain understudied.

Spatial projections under climate change scenarios suggest changing zones of risk for *Leptospira* [41, 42]. However, the effect of temperature on the distribution of zoonoses and the risk of incursion with future climate change remains a key gap. The limited spatial projections provided by included studies, as well as their lack of focus on future incursion risk, was unexpected given the significant economic and agricultural costs that can result from zoonotic incursions [82]. Furthermore, the lack of evidence for the potential changing distribution of zoonoses under climate change scenarios limits understanding of the strength of these associations and the risk of incursion. Recent experiences with COVID-19 and monkeypox highlight the importance of preparing for disease incursions that evade country boundaries. Predominantly, studies included in this review focused on the northern hemisphere countries, many of which have surveillance systems and resources for detection. Therefore, while understanding how biosecurity risks from zoonotic pathogens will change under future climate conditions is an urgent need, documenting these patterns in countries with few resources is critical. A One Health approach to biosecurity that acknowledges and builds on the interconnected relationship between human, animal, plant, and environmental health [83, 84] provides a starting point to prioritise and assess the risk of incursions from zoonotic pathogens in a changing climate. Importantly, a regional focus that spans multiple nations will be necessary to prepare for zoonotic pandemics in a changing climate.

## 5. MeSH Terms

Climate Change, Global Burden of Disease, One Health, Public Health, Zoonoses.

## Figures and Tables

**Table 1:** Inclusion and exclusion criteria.

Inclusion criteria	Exclusion criteria
Peer-reviewed journal article	Not a peer-reviewed journal article
Written in English	Not written in English
Original study	Not an original study (e.g., narrative literature reviews or other systematic reviews)
Available in full text	Not available in full text
Articles focused on temperature as a climate variable	Articles did not focus on temperature as a climate variable
Articles considered the impact of temperature on a zoonotic pathogen in terms of incursion, distributional burden (e.g., temporal or spatial distribution) or risk of such events	Articles did not consider the impact of temperature on a zoonotic pathogen in terms of incursion, distributional burden (e.g., temporal or spatial distribution) or risk of such events
Articles focused on a zoonotic pathogen in livestock or wildlife population/s of mammalian vertebrates that can be transmitted indirectly, and for which the transmission pathway does not involve a nonmammalian and nonvertebrate intermediate host.	Articles did not focus on a zoonotic pathogen in livestock or wildlife population/s of mammalian vertebrates that can be transmitted indirectly, and for which the transmission pathway does not involve a nonmammalian and nonvertebrate intermediate host.

**Table 2:** Key findings of temperature effects on zoonotic pathogens.

	Study	Study population	Measurement of temperature	Zoonotic pathogen and measure	Temperature effects
Parasites	Miterpáková et al. [29] The Slovak Republic	Red foxes (*n* = 3096), 2000–2004	Mean annual air temperature (2000–2004)	*Echinococcus multilocularis* Prevalence and worm burden	(i) Lower mean temperature was associated with greater prevalence of *E. multicocularis*
	Dybing et al. [30] Western Australia	Red foxes (*n* = 147), February and March 2010	5-year mean, minimum and maximum temperature averages	*Uncinaria stenocephala*, *Toxocara canis* and *Taenia serialis* Prevalence and infection intensity	(i) Positive association between *U. stenocephala* and 5-year average minimum temperature (*p* < 0.001)
	Tolnai et al. [31] Hungary	Red foxes (*n* = 1612), November 2008–Febuary 2009 and November 2012–February 2013	Mean annual temperature (2008 and 2012)	*Echinococcus multilocularis* Prevalence and worm burden	(i) Inverse association between mean temperature and infection intensity for *E. multicocularis* (95% CI OR = 0.492–0.818)
	De Chaneet, and Dunsmore [32] South-Western Australia	Sheep (*n* = 221), August 1982-November 1984	Mean monthly minimum and maximum temperatures (1982–1984)	*Trichostrongylus spp* Prevalence	(i) Inverse association between *T. vitrinus* and mean autumn (*r* = −0.48, *p* < 0.001), winter (*r* = −0.49, *p* < 0.001) and spring (*r* = −0.48, *p* < 0.001) temperatures. (ii) Positive association between *T. colubriformis* and mean autumn (*r* = 0.56, *p* < 0.001), winter (*r* = 0.58, *p* < 0.001) and spring (*r* = 0.45, *p* < 0.01) temperatures.
	Pilarczyk et al. [33] Lubuskie voivodeship	Mouflons (*n* = 40), 3 years	Monthly temperature (mean, minimum and maximum) (3 years)	*Strongyloides sp*, *Capillaria sp* and *Trichostrongylidae* Prevalence and infection intensity	(i) Positive association between protozoan infection and temperature (*r* = 0.43, *p* < 0.001) but didn't differentiate zoonotic protozoans. (ii) For the included zoonotic pathogens (Strongyloides sp, Trichostrongylidae and Capillaria sp.) the results were nonsignificant.
	McMahon et al. [34] Northern Ireland	Sheep (*n* = ), 1999–2009	Mean monthly temperature variables and soil temperature (1999–2009)	Trichostrongylus (*trichostrongylosis*, *teldorsagiosis and haemonchosis*) and strongyloidosis Prevalence	(i) Temperature increasing earlier and more significantly in February and the spring months of March to May. (ii) No correlation between temperature and the diagnostic rate for *trichostrongylosis* and strongyloidosis.
	Tolnai et al. [35] Hungary	Red foxes (*n* = 3,304) and wild boars (*n* = 0.29 million), 2006–2013	Mean annual temperature variables (2006–2013)	*Trichinella spiralis* and *Trichinella britovi* Prevalence	(i) No correlation between environmental parameters and *T. spiralis* (ii) Inverse association: mean temperature and *T. britovi* spatial distribution (95% CI of OR: 0.477–0.832).

Bacteria	Patterson et al. [36] California	Livestock (cattle, goats, pigs and sheep) (*n* = 558 faecal samples from 16 farms), 2015–2016	Daily temperature variables (2015–2016)	Shigella toxin-producing *E. coli* (STEC) Prevalence	(i) Inverse association: for every increase in daily maximum temperature, odds of STEC-positive sample decreased (OR: 0.95; 95% CI 0.91–0.98)
	Gao et al. [37] Mainland China	Pigs (*n* = 18,862 outbreaks), 2006–2014	Monthly temperature variables (2006–2014)	*Pasteurella multocida* Prevalence and number of outbreaks	(i) Positive association between mean monthly temperature and prevalence of *P. multocida*. (ii) Results of multivariate analysis for temperature with lag of 0 months and 1 month: *r* = 0.076 (95% CI 0.021–0.131, *p* < 0.001) and *r* = 0.732 (95% CI 0.65–0.814, *p* < 0.001)
	Vogt et al. [38] Southern Ontario	Raccoons (*n* = 627), 2011–2013	Mean temperature of previous 30 and 14 days (2011–2013)	*Campylobacter jejuni* Prevalence	(i) Inverse association between *C. jejuni* prevalence and temperature (OR for medium temperature with low as the reference was 0.40, 95% CI 0.16–0.98).
	Nsoh et al. [39] Northern Region of Ghana	Cattle (*n* = 131), sheep (*n* = 44), goats (*n* = 15), pigs (*n* = 562) and humans (*n* = 6), January-December 31st 2003	Monthly temperature variables (January–December 31st 2003)	*Anthracis bacillus* Prevalence	(i) All high risk areas (i.e., where prevalence was greater) experienced medium, high or very high temperatures
	Deka et al. [40] Global (with additional circum-polar focus)	Livestock animals and humans (*n* = 872), 1954–2021	Mean maximum and minimum land-surface temperature (1954–2021) and mean 8-day temperature	*Anthracis bacillus* Prevalence	(i) Two principal component analysis models (PC 1 and PC 2) for temperature. Contribution of climate conditions (including temperature) = 10.25% (global model) and 10.03% (PC 1; 10.33% = PC 2) (Circum-polar model) (ii) In PC 1, land surface temperature contributed 22.69% in the global model and 12.087% in the circumpolar model In PC 2, land surface temperature contributed 16.58% to the global model
	Zakharova et al. [41] Russian Arctic	Livestock (predominantly cattle and horses) (*n* = 808), 2000–2019	Mean annual air temperature (2000–2019)	*Leptospira* Prevalence and future risk projections	(i) Contribution of the following factors to the analysis model for *leptospira*: (ii) Mean yearly air temperature = 4.97 (iii) Mean yearly number of days with the air temperature above 0 degrees Celsius = 4.45 (iv) Mean yearly amplitude of daily air temperature = 4.04 (v) Temperature ∴ accounted for ∼12% of the entire model
	Zakharova et al. [42] Republic of Sakha (Yukutia), Russian Federation	Predominantly livestock (cattle, horses, pigs and sheep), as well as some wild animals (reindeer and rabbits) and domestic cats and dogs (*n* = 2,728), 1995–2019	Mean temperature variable (including monthly, wettest quarter, warmest quarter) (1995–2019)	*Leptospira* Prevalence and future risk projections	(i) Contribution of the following factors to the analysis models for *Leptospira* (ii) Mean temperature of the wettest quarter = 10.3% (iii) Temperature seasonality = 5.2% (iv) Temperature variation/range = 13.2%
	Mendes et al. [43] England	Pigs (*n* = 251,3973), 2014	Daily air temperature (2014)	*Ascaris suum* Prevalence	(i) Positive association between *A. suum* prevalence and temperature (*β* = 0.007; 95% CI = 0.003, *p* = 0.012)
	Wang et al. [44] China	Pigs (*n* = 281,967), 2008–2018	Monthly temperature (extremes and averages) (2008–2018)	*Erysipelothrix rhusiopathiae* Prevalence	(i) Positive association between extreme maximum temperature and prevalence of *E. rhusiopathiae* for hotspots (i.e., where prevalence was high) (OR = 1.143, 95% CI 1.032–1.342)

Fungi	Cafarchia et.al. [45] Southern Italy	Rabbits (*n* = 810), October 2006–Febuary 2007	Daily mean temperature variables (October 2006–Febuary 2007)	Dermatophytes (including *Trichophyton mentagrophytes* and *Microsporum canis*) Prevalence	(i) Positive association: dermatophyte prevalence was significantly higher in holding areas with high temperature (>20 degrees Celsius, *p* < 0.05) associated with humidity ranging from 62–65%.

**Table 3:** Summary of the associations found between temperature and pathogen prevalence.

	Positive	Inverse
Parasites	*Uncinaria stenocephala* *Trichostrongylus columbriformis*	*Echinococcus multicocularis* *Trichostrongylus vitrinus* *Trichinella britovi*

Bacteria	*Ascaris suum* *Pasteurella multocida* *Anthracis bacillus* *Erysipelothrix rhusiopathiae* *Leptospira*	Shigella toxin-producing *E. coli* *Campylobacter jejuni*

Fungi	*Dermatophytes* (including *Trichophyton mentagrophytes* and *Microsporum canis*)	

**Note:** Associations for *Strongyloides sp*, *Trichostrongylidae*, *Capillaria sp* and *Trichinella spiralis* were not statistically significant [33, 34]

## Data Availability

Additional data that supports the findings of this study are available in the supplementary material.

## Abbreviations

JEV: Japanese encephalitis virus
PRISMA: Preferred Reporting Items for Systematic Review and Meta-Analyses
MeSH: Medical subject headings
STEC: Shigella toxin-producing *E. coli*.

## Supplementary Materials

Supplementary Materials Appendix S1: search terms used in PubMed, Scopus, and Web of Science. Appendix S2: search strategy applied to the grey literature. Appendix S3: the categories used in data extraction of the included studies. Data were extracted for all applicable categories listed. Appendix S4: summary table of the critical appraisal of the included studies using the Johanna Briggs Institute Critical Appraisal Tool for cross-section studies. Appendix S5: summary table of additional data extracted from included studies included information on spatial variation of zoonotic pathogens, the measure and impact/effect of climatic variables other than temperature, the measure and impact/effect of nonclimatic factors, and key limitations of the studies. Extracted data are categorised by pathogen type.
