# Supplementary material for: The Effect of Temperature on the Distribution of Zoonotic Pathogens in Livestock and Wildlife Populations: A Systematic Review
Source: Transbound Emerg Dis. 2023 Aug 18;2023:2714539. doi: 10.1155/2023/2714539 (PMC12017053; doi:10.1155/2023/2714539)
Supplement: Supplementary Materials — Appendix S1: search terms used in PubMed, Scopus, and Web of Science. Appendix S2: search strategy applied to the grey literature. Appendix S3: the categories used in data extraction of the included studies. Data were extracted for all applicable categories listed. Appendix S4: summary table of the critical appraisal of the included studies using the Johanna Briggs Institute Critical Appraisal Tool for cross-section studies. Appendix S5: summary table of additional data extracted from included studies included information on spatial variation of zoonotic pathogens, the measure and impact/effect of climatic variables other than temperature, the measure and impact/effect of nonclimatic factors, and key limitations of the studies. Extracted data are categorised by pathogen type. [file 2714539.f1.docx]

**Appendices**

**Appendix 1: Search terms**

**PubMed**

((((Climat*[Title/Abstract] OR Environment*[Title/Abstract]) AND (Temperature[Title/Abstract])) AND (Animal*[Title/Abstract] OR Human*[Title/Abstract] OR Livestock[Title/Abstract] OR Wildlife[Title/Abstract])) AND (Incur*[Title/Abstract] OR Introduc*[Title/Abstract] OR Distribut*[Title/Abstract] OR Risk[Title/Abstract])) AND (Zoono*[Title/Abstract] OR Pathogen*[Title/Abstract] OR Disease*[Title/Abstract] OR Illness*[Title/Abstract] OR Infect*[Title/Abstract])

**Scopus**

TITLE-ABS-KEY(climat* OR evironment*) AND TITLE-ABS-KEY(animal* OR human* OR livestock OR wildlife) AND TITLE-ABS-KEY(incur* OR distribut* OR risk OR introduc* ) AND TITLE-ABS-KEY(zoono* OR pathogen* OR disease* OR illness* OR infect*) AND TITLE-ABS-KEY(temperature)

**Web of Science**

((((AB=((Climate* OR Environment*))) AND AB=((Temperature))) AND AB=((Animal* OR Livestock OR Wildlife OR Human*))) AND AB=((Incur* OR Introduc* OR Distribut* OR Risk))) AND AB=((Zoonotic* OR Pathogen* OR Disease* OR Illness* OR Infect*))

**Note:** Only Scopus allowed screening of title, abstract and keywords at the same time and therefore the equivalent filter in the other databases (either title/abstract or just abstract) was used.

**Appendix 2: Grey literature search strategy**

The grey literature search was conducted using the following sources: World Health Organisation, Food and Agricultural Organisation of the United Nations, World Organisation for Animal Health, Centre for Disease Control and Prevention, European Centre for Disease Prevention and Control, and government institutions such as the Australian Government Department of Health and Aged Care and the Australian Department of Agriculture, Fisheries and Forestries.

Grey literature search terms:

- Animal health
- OneHealth
- Zoonotic
- Global warming
- Incursion

These terms were searched in combined form using ‘AND’.

**Appendix 3: Template categories used for data extraction**

- Author
- Publication date
- Location/region studied
- Study aim
- Pathogen/s studies
- Affected species studied
- Pathway/mode of transmission
- Measurement of infection or pathogen (e.g., how cases of disease were determined)
  - Confidence interval/s for measurement
- Measurement of temperature (e.g., daily, annually or monthly)
- Total duration of included temperature data (i.e., number of years of data included)
- Impact/effect of temperature (past, current and/or predicted)
  - Confidence interval for included effect of temperature
- Other climactic variables considered (e.g., precipitation)
- Total duration of included climactic data (i.e., number of years of data included)
- Non-climatic factors assessed
- Author’s conclusion/s
- Limitations
- Confounders

**Appendix 4: Johanna Briggs Institute Critical Appraisal Tool (Cross-sectional Studies)**

|  | Were the criteria for inclusion in the sample clearly defined? | Were the study subjects and the setting described in detail? | Was the exposure measured in a valid and reliable way? | Were objective, standard criteria used for measurement of the condition? | Were confounding factors identified? | Were strategies to deal with confounding factors stated? | Were the outcomes measured in a valid and reliable way? | Was appropriate statistical analysis used? | Overall appraisal | Comments (including reason for exclusion) |
| --- | --- | --- | --- | --- | --- | --- | --- | --- | --- | --- |
| **Miterpakavoa, M., Dubinsky, P., Reiterova, K., & Stank, M. (2006) (27)** | Yes | Yes | Yes | Yes | No | N/A | Yes | Yes | Included |  |
| **Dybing, N, A., Fleming P, A., & Adams, P, J. (2013) (28)** | Yes | Yes | Yes | Yes | No | N/A | Yes | Yes | Included |  |
| **Tolnai, Z., Szell, Z., & Sreter, T. (2013) (29)** | Yes | Yes | Yes | Yes | Yes | Yes | Yes | Yes | Included |  |
| **De Chaneet, G,C., & Dunsmore, J, D. (1988) (30)** | Yes | Yes | Yes | Yes | Yes | Yes | Yes | Yes | Included |  |
| **Pilarczyk, B., et al. (2022) (31)** | Yes | Yes | Yes | Yes | No | N/A | Yes | Yes | Included |  |
| **McMahon, C., et al. (2012) (32)** | Yes | Yes | Yes | Yes | No | N/A | Yes | Yes | Included |  |
| **Tolnai, Z., Szell, Z., Marucci, G., Pozio, E., & Sreter, T. (2014) (33)** | Yes | Yes | Yes | Yes | Yes | Yes | Yes | Yes | Included | Only one farm included in analysis (under 500 samples). Sample size may be too limited. |
| **Patterson, L., Navarro-Gonzalez, N., Jay-Russel, M, T., Aminabadi, P., & Pires, A, F, A. (2022) (34)** | Yes | Yes | Yes | Yes | No | N/A | Yes | Yes | Included |  |
|  | Were the criteria for inclusion in the sample clearly defined? | Were the study subjects and the setting described in detail? | Was the exposure measured in a valid and reliable way? | Were objective, standard criteria used for measurement of the condition? | Were confounding factors identified? | Were strategies to deal with confounding factors stated? | Were the outcomes measured in a valid and reliable way? | Was appropriate statistical analysis used? | Overall appraisal | Comments (including reason for exclusion) |
| **Gao, X., Xiao, J., Qin, H., Cao, Z., & Wang, H. (2016) (35)** | Yes | Yes | Yes | Yes | No | N/A | Yes | Yes | Included |  |
| **Vogt, N, A., et al. (2020) (36)** | Yes | Yes | Yes | Yes | No | N/A | Yes | Yes | Included |  |
| **Nsoh, A, E., et al. (2016) (37)** | Yes | Yes | Yes | Yes | No | N/A | Yes | Yes | Included |  |
| **Deka, M, A., Veira, A, R., & Bower, W, A. (2022) (38)** | Yes | Yes | Yes | Yes | Yes | Yes | Yes | Yes | Included |  |
| **Zakharova, O, I., et al. (2021) (39)** | Yes | Yes | Yes | Yes | No | N/A | Yes | Yes | Included |  |
| **Zakharova, O, L. (2020) (40)** | Yes | Yes | Yes | Yes | No | N/A | Yes | Yes | Included |  |
| **Mendes, A,J., Ribeiro, A, I., Severo, M., & Niza-Ribeiro. (2017) (41)** | Yes | Yes | Yes | Yes | No | N/A | Yes | Yes | Included |  |
| **Wang et al., (2020) (42)** | Yes | Yes | Yes | Yes | Yes | Yes | Yes | Yes | Included |  |
| **Cafarchia, et.al. (2010) (43)** | Yes | Yes | Yes | Yes | No | N/A | Yes | Yes | Included |  |

| **Pathogen type**  **Appendix 5: Additional extracted data from included studies** | **Study** | **Study design** | **Spatial variation** | **Measure and impact/effect of other climatic variables** | **Measure and impact/effect of non-climatic factors** | **Study limitations** |
| --- | --- | --- | --- | --- | --- | --- |
| **Parasites** | **Miterpakavoa, M., Dubinsky, P., Reiterova, K., & Stank, M. (2006) (27)** | Cross-sectional (with modelling components). | Increase in prevalence in 2000-2002 - greatest in northern regions.  Prevalence and worm burden increased in the second half of 2004 - greatest prevalence in northeastern Slovakia. | Mean annual precipitation (2000-2004)  Significant correlation between prevalence of the tapeworm in red foxes and mean annual precipitation (r = 0.933, p = 0.021). Mean annual precipitation was also significantly correlated with worm burden in red foxes (r = 0.973, p = 0.020). | Relative density of small mammals was significantly associated with prevalence of the tapeworm in red foxes (r = 0.709, p = 0.022). There was also a strong relationship between density and worm burden. | The measurement of the population density of small mammals (intermediate hosts) has only been performed in a few areas and there has been no blanket monitoring in Slovakia. |
|  | **Study** | **Study design** | **Spatial variation** | **Measure and impact/effect of other climatic variables** | **Measure and impact/effect of non-climatic factors** | **Study limitations** |
|  | **Dybing, N, A., Fleming P, A., & Adams, P, J. (2013) (28)** | Cross-sectional (with modelling components). | N/A | Average relative humidity for previous six months and presence of native or other vegetation (% cover)  There was a significant association between environmental measures and the presence of four of the five most prevalent parasite species. Sites with *U. stenocephala* were generally more humid, had warmer minimum temperatures (P<0.001), and more native vegetation. Prevalence of *U. stenocephala* was 18.2% and infection intensity was 17 (SD 17.33). Sites with greater percentage prevalence of T. canis generally had more native vegetation. | Sites with greater prevalence of *T. canis* and *U. stenocephala* generally had more native vegetation. | Only based in Western Australia. Lacks explanatory power. |
|  | **Tolnai, Z., Szell, Z., & Sreter, T. (2013) (29)** | Cross-sectional (with modelling components). | Prevalence was highly clumped - 0-38.2% in northern counties vs 0-13.7% in other counties | Mean annual precipitation (2008 and 2012)  Whilst spreading and emergence of *E. multilocularis* was observed in Hungary before 2009, a non-significant decrease of infection prevalence and intensity was noted between the two collection periods which was likely due to the considerably different mean annual precipitation in 2007-2008 and 2001-2012 (541-572mm vs 382-440mm). | There was no association between soil water retention or soil permeability in the home range of foxes and E. multicocularis infection intensity. Permanent water bodies were significantly more frequently detected in the home range of *E. multicocularis* infected foxes compared to uninfected foxes but there was a lack of correlation with the area of buffer zones of water. | A potential limiting or biasing factor might relate to the red foxes being those successfully killed by hunters as part of a pre-existing rabies immunisation and control programme. The success of this programme could differ between counties and maybe this could account for some of the clustered distribution. However, authors do not list any limitations. |
|  | **Study** | **Study design** | **Spatial variation** | **Measure and impact/effect of other climatic variables** | **Measure and impact/effect of non-climatic factors** | **Study limitations** |
|  | **De Chaneet, G,C., & Dunsmore, J, D. (1988) (30)** | Cross-sectional (with modelling components). | No significant difference in prevalence across localities based on mean summer temperature, but there were significant differences based on temperature of all other seasons. | Mean annual rainfall (1982-1984)  Rainfall only varied systematically with categories based on mean spring temperature. There were no significant correlations between prevalence of either *T. vitrinus* or *T. colubriformis* and rainfall and length of growing season. However, the prevalence of *T. rugatus* was inversely associated with rainfall and length of growing season. Their analysis of categorised data showed a significant effect of rainfall on the prevalence of *T. rugatus* and *T. colubriformis* and a seasonal effect on the prevalence of *T. vitrinus*. | N/A | Limited due to potential confounding relationship. It was also not clear whether or not seasonal changes in prevalence of individual species was present and multivariate analysis methods would have been usefully in more clearly defining these relationships. |
|  | **Pilarczyk, B., et al. (2022) (31)** | Cross-sectional (with modelling components). | N/A | Precipitation range and averages during experimental period (3-years)  The intensity of protozoan infection was significantly correlated (positively) with precipitation, however, this correlation was week (r = 0.17) and did not differentiate zoonotic protozoans. | N/A | Many different parasites studied and there is not a distinction made between those that require intermediate hosts or can be transmitted to humans. The transmission pathways and how temperature effects prevalence and intensity are not well explained. Only one farm included in the study so the generalisability of the results may be limited. |
|  | **Study** | **Study design** | **Spatial variation** | **Measure and impact/effect of other climatic variables** | **Measure and impact/effect of non-climatic factors** | **Study limitations** |
|  | **McMahon, C., et al. (2012) (32)** | Cross-sectional (with modelling components). | Regions 1 and 2 - peak in trichostrongylosis between August and October and two smaller peaks in February and May.  All regions - high case numbers of strongyloidosis recorded in August, September and October and secondary peaks observed in March and April (Region 1), February and May (Region 2) and April (Region 4). | Rainfall (mm) per month (1999-2009)  The observed increase rainfall over the study period was suggested by the authors to be sufficient to slow the desiccation of faecal deposits which typically results in the death of eggs and pre-infective larvae. | Authors did not sufficiently investigate the potential spread of anthelmintic resistance as a determinant for the increased incidence of nematode parasitic infections observed. They suggested that it is unclear whether drug resistance (if it is even present) may represent a more important determinant than climate change with respect to the increasing incidence. | Did not measure anthelmintic resistance so authors suggestions on the matter are limited and not grounded in strong evidentiary support. |
|  | **Tolnai, Z., Szell, Z., Marucci, G., Pozio, E., & Sreter, T. (2014) (33)** | Cross-sectional study with modelling components, including geographical information systems (GIS) analysis. | N/A | Mean annual precipitation (2006-2013)  No correlation between environmental parameters and T. spiralis was found. Positive correlation between annual precipitation and T. britovi in foxes (P<0.05) and wild boars (P<0.005) which was not confirmed by multiple regression analysis and logistic regression analysis. | There was no correlation between environmental parameters and T. spiralis worm burden (LPG). There was no correlation between protected areas, permanent water bodies, soil water retention or soil permeability in the home range of foxes and wild boars and the T. britovi LPG. Inverse correlation between agricultural areas of the home range of foxes (P<0.0001) or of foxes and wild boars (P<00001) for T. britovi. Positive correlation between non-agricultural areas of the fox home range for T. britovi (P<0.0001) was observed. | Potential limitation was the lack of data identified for *T. spiralis*. A significant number of animals were tested but a greater number may have aided analysis or demonstrated an appreciable affect for *T. spiralis.* |
|  | **Study** | **Study design** | **Spatial variation** | **Measure and impact/effect of other climatic variables** | **Measure and impact/effect of non-climatic factors** | **Study limitations** |
| **Bacteria** | **Patterson, L., Navarro-Gonzalez, N., Jay-Russel, M, T., Aminabadi, P., & Pires, A, F, A. (2022) (34)** | Cross-sectional (with modelling components). | N/A | No other climate factors included in final modelling analysis. | The odds ratios differed between livestock species and the odds of STEC increased 6.23 times for farms that housed multiple livestock species within the same barn compared to farms that housed them separately. The odds of a positive STEC sample were more than three times greater for a farm that allowed livestock contact with wild areas. | There were contradictions with other studies that used other methodology relating to time of sampling. The temperatures measured in this area are also higher than what is often observed in other states and therefore may not be generalisable. The final model also did not appear to consider precipitation and/or humidity which limits understanding and interpretation of the results, as well as potential identification of confounding. |
|  | **Gao, X., Xiao, J., Qin, H., Cao, Z., & Wang, H. (2016) (35)** | Cross-sectional (with modelling components including cluster analysis). | Approx. 75% of cases originated from six provinces of the hot region of Mainland China (e.g., south central Mainland China)  Few cases in the other 25 provinces | Mean monthly rainfall, relative humidity and average windspeed (January 2006-December 2014)  Cross-correlation analysis with meteorological factors revealed that there was a statistically significant negative correlation between average wind speed and the number of cases that month. The current month's average windspeed inversely correlated with the number of cases. | N/A | Could not determine the sensitivity of disease detection for the study data. It is possible that differential diagnosis may have been an issue without laboratory confirmation in some cases given the similarity of pasteurellosis to other diseases such as swine toxoplasmosis. Farmers may also have understated cases. |
|  | **Study** | **Study design** | **Spatial variation** | **Measure and impact/effect of other climatic variables** | **Measure and impact/effect of non-climatic factors** | **Study limitations** |
|  | **Vogt, N, A., et al. (2020) (36)** | Cross-sectional (with modelling components). |  | Total rainfall for previous 14 days and previous 30 days (2011-2013)  In the case of the interaction between rainfall and year, the following comparisons were associated with a significantly greater odds of isolating *C. jejuni*, but only in 2013: medium vs low, high vs low. In 2012, a high total rainfall was associated with a significantly greater odds of isolating *C. jejuni* compared to medium total rainfall. | N/A | Farms and conservation areas were not differentiated in the final model. Only two time periods were measured for the impact of rainfall and temperature, and it is possible that other measurements of these variables may have more accurately predicted association. Some predictors may also have been unmeasured and were therefore missing from the model. This was suggested due to the inconsistencies observed across the years. There was also likely some bias due to the sampling method involving live baiting (i.e., some raccoons may have been "trap-happy" and have been caught/counted multiple times). |
|  | **Nsoh, A, E., et al. (2016) (37)** | Cross-sectional (with modelling components). | Eastern corridor of the Northern region was deemed a hot spot area.  High risk areas: East Mamprusi, Bunkpurugu-yunyoo, Gushiegu, Karaga, Yendi, Saboba-Chereponi, Tamale Municipal, East Gonja, Nanumba North and South and Zabzugu-Tatale | Rainfall (2003-2012)  During the study period, some districts such as Yendi experienced very high rainfall and might have experienced flooding and erosions. Eight out of the other twenty districts including Savelugu-Nanton experienced low rainfall. | Areas with low soil pH, medium soil pH and high soil pH were recorded. Districts such as Bunkpurugu-yunyoo even recorded soil pH that was very high. The finding that areas with the soil pH districts such as Yendi were more suitable for anthrax spore survival. This is supported by other studies that find pH above 6.1 to alkaline is an important determinant of increased spore survival. | Only used mean temperature and mean rainfall which may not capture sufficient detail to determine the role of temperature on outbreaks in these areas. |
|  | **Study** | **Study design** | **Spatial variation** | **Measure and impact/effect of other climatic variables** | **Measure and impact/effect of non-climatic factors** | **Study limitations** |
|  | **Deka, M, A., Veira, A, R., & Bower, W, A. (2022) (38)** | Cross-sectional study with ensemble ecological niche modelling. | Risk areas for livestock: Canadian Plains, the United States, much of Central America (including Columbia, Venezuela, Peru and Ecuador), northern Argentina, northeastern Brazil, Central Valley region of Chile, much of Africa and Eurasia, and many regions in East Asia, Indonesia and Australia. | Precipitation, vapour pressure, wind speed, climate water deficit, amongst other variables (1981-2010)  Variable importance under the global model was highest for vegetation and land surface temperature, soil characteristics, primary climate conditions (including maximum temperature and minimum temperature) and topography. Variable importance under the circumpolar model was highest for soil characteristics, topography, derived climate, vegetation and land surface temperature, as well as the primary climate characteristics. | N/A | Results may have been biased by the under-reporting, under-recognition and concern regarding anthrax in regions where anthrax is not endemic. Overprediction was evident in much of Northern Australia in the global model, as well as the Great Lakes regions of the United States and Canada. The modelling of livestock risk also does not account for different livestock management practices (e.g., vaccination rates, livestock biosecurity and herd managements). |
|  | **Zakharova, O, I., et al. (2021) (39)** | Cross-sectional (with modelling components applying Forest-based Classification). | Climatic Projections for 2100 = increase in leptospirosis risk in most of the area studied (greatest in the northern part of European Russia and Western Siberia)  In certain areas, the climate-dependent risk of leptospirosis was predicted to increase more than four-fold. | Precipitation and seasonality (CMIP5 models used for future projections)  The calculation of the seasonality index demonstrated prevalence of cases in March as S = 1.53, in June as S = 2.27, and in August as S = 1.73. Yearly precipitation for the period with the air temperature above 0 degrees celsius was given 5.04 for importance in the model (approx. 4%). The importance values of 4.52 (approx. 4%) and 2.7 (approx. 2%) were awarded to yearly precipitation and yearly precipitation for the period with the air temperature below 0 degrees celsius respectively. | The results indicated that socioeconomic factors were of greatest importance for explaining the observed distribution of leptospirosis cases. Population density contributed approximately 21% to the model, followed by proportion of crop area in the total area of the region (14%), livestock unit density index (13%) and budgetary investments into the development of agriculture per unit area (11%). The climate variables then ranked in importance along with landscape factors. | There were a limited number of spatial units available for analysis and the Forest-based Classification and Regression tool performs better with larger datasets. There may also have been uncertainty regarding the binding of specific values to a territorial unit. The socio-economic factors may therefore have inadequately reflected the true significance of the factor in the places with registration of leptospirosis. The forecast for the future is based only on climatic factors and not on the socio-economic determinants that demonstrated a higher contribution to the model applied. |
|  | **Study** | **Study design** | **Spatial variation** | **Measure and impact/effect of other climatic variables** | **Measure and impact/effect of non-climatic factors** | **Study limitations** |
|  | **Zakharova, O, L. (2020) (40)** | Cross-sectional (with modelling components applying MaxEnt). | High and medium risk zones = southeastern part of RSY (under current and future climate conditions)  Under current climate conditions, high risk zones include Namsky, Ust-Alansky, Tattinsky, Alekseevsky, Churapchinsky districts, and the city of Yakutsk.  Projected expansion of leptospirosis under future climate conditions. Additional high-risk zones include the Medino-Kangalassky, Amingsky, and Ust-May districts  Some areas in the north and northeast of the Republic demonstrate a statistically significant reduction in suitability | BIOCLIM variables used e.g., average monthly precipitation (1970-2000 and INMCM4 climate data used for future projections)  Maximum suitability for transmission was in areas with mean temperature of the wettest quarter being approximately 14 degrees celsius and above, low altitudes (less than 500m above sea level), considerable variation in the monthly temperatures within a year (10 degrees celsius and above), pronounced seasonal variation (+/- 20 degrees celsius), relatively low precipitation of approximately 20-60mm/month, relatively high cattle density, and soil pH of 5.5 and above. | The land cover categories most closely associated with the risk of leptospirosis were urban and built-up areas, open deciduous needleleaf forest, open ground and rock outcrops, coastal vegetation, permanent wetlands, and grasslands. Distance to the nearest water body was inversely associated with leptospirosis which suggested that most cases occurred no farther than 2500m from water. Cattle density was significantly associated with recorded outbreaks, and this was used as a proxy for human presence and economic activity as well as an indicator of the cattle population’s exposure to leptospires. | The study is not directly comparable to other studies as the variables used are somewhat different. The choice of final variables included in the model were based on methodology to reduce multicollinearity, and this may have meant that some more general variables (e.g., yearly mean temperature and precipitation) were excluded. |
|  | **Mendes, A,J., Ribeiro, A, I., Severo, M., & Niza-Ribeiro. (2017) (41)** | Cross-sectional (with modelling components including Gaussian multilevel linear mixed modelling and building chloropleth maps). | Prevalence in farms in southwest (4.8%; n = 305,742; excluding Wales), southeast (4.4%; n = 867,017) and north (4.2%; n = 1,341,214) | Monthly relative humidity and daily rainfall totals (2014)  A positive association between the percentage of infected animals per batch and both the relative humidity and rainfall was observed. | The farms located in areas with the best ranks of socioeconomic deprivation had a significantly lower proportion of porcine ascariasis. With an increase in the number of animals sent to slaughter, the proportion of porcine ascariasis decreased significantly. The farms in areas with coarse soils had a significantly higher percentage of infected animals compared to medium texture soil class and peat soils. | The extent to which the study can be generalised to the population of English farms may be limited. According to the authors, the methodology of diagnosis through post-mortem inspection applied in this study is novel and has never been used in the context of porcine ascariasis. The inclusion of postcode may have also introduced information bias since rural areas are less accurately reported. |
|  | **Study** | **Study design** | **Spatial variation** | **Measure and impact/effect of other climatic variables** | **Measure and impact/effect of non-climatic factors** | **Study limitations** |
|  | **Wang et al., (2020) (42)** | Cross-sectional (with modelling components including cluster analysis). | • Swine erysipelas outbreaks concentrated in South Central China (accounted for 77% of outbreaks in 2008-2018). • Coldspots in Northeast Mainland China disapeared since 2013 • Potential outbreak areas in Northeast Mainland China (Heilongjiang, Jilin, Liaoning, Beijing, Tianjin, Hebei and Shandong) | Precipitation, water vapour pressure, wind speed, humidity and sunshine (2008-2018) For hotspots, extreme maximum temperature (OR 1.143), total precipitation (2.298) and daily precipitation equal to or greater than 0.1mm (OR = 2.396) exhibited positive associations. Maximum wind speed and concentration of certain pollutants demonstrated an inverse association. In potential outbreak areas, only precipitation was positively associated to a significant extent with swine erysipelas. | N/A | Analysis of seasonality in potential outbreak areas was limited by lower case numbers. |
| **Fungi** | **Cafarchia, et.al. (2010) (43)** | Cross-sectional (with modelling components). | N/A | Relative daily humidity (October 2006 to February 2007)  Dermatophyte prevalence was significantly higher in holding areas with high temperature (>20 degrees celsius) associated with humidity ranging from 62-65%. In holdings that were kept at temperatures <20 degrees celsius, there was a trend towards linear increases in the prevalence of dermatophyte infection (not significant) according to the humidity increasing. | N/A | Small time period of study that cannot provide data on seasonal variation of different time periods. |
